# Gender-specific factors contributing to visceral obesity including the sleep-obesity relationship: a large-scale cross-sectional study from East Asia

**DOI:** 10.1038/s41598-022-24863-6

**Published:** 2022-11-24

**Authors:** Katsuki Saito, Takeshi Shimamoto, Yu Takahashi, Kazuya Okushin, Mami Takahashi, Yukari Masuda, Takako Nishikawa, Naomi Kakushima, Ryoichi Wada, Nobutake Yamamichi

**Affiliations:** 1 Faculty of Medicine, The University of Tokyo, 7-3-1, Hongo, Bunkyo-ku, Tokyo, Japan; 2 Kameda Medical Center Makuhari, CD-2, 1-3, Nakase, Mihama-ku, Chiba, Japan; 3 Department of Gastroenterology, Graduate School of Medicine, The University of Tokyo, 7-3-1, Hongo, Bunkyo-ku, Tokyo, Japan; 4 Center for Epidemiology and Preventive Medicine, The University of Tokyo Hospital, 7-3-1, Hongo, Bunkyo-ku, Tokyo, Japan

**Keywords:** Diseases, Medical research, Risk factors

## Abstract

Our study aimed to evaluate the relationship between visceral obesity and its associated factors, especially sleep duration in East Asia. We conducted univariate and multivariate analyses using the data of 2538 participants (mean age 56.4 ± 10.8 years) who underwent medical checkups and computed tomography of the abdomen to calculate the visceral fat area from 2008 to 2020. We additionally performed logistic regression analyses using each sleep-duration group (< 5, 5–6, 6–7, 7–8, and ≥ 8 h) and their respective propensity scores as covariates. According to the criteria of visceral obesity(a visceral fat area ≥ 100 cm^2^), 1147 of 1918 men (59.8%) and 131 of 620 women (21.1%) had visceral obesity. In multivariate analyses, visceral obesity was significantly associated with age, body mass index and triglyceride in both genders, high-density lipoproteins, uric acid levels, and daily alcohol consumption in men; and glycated hemoglobin (HbA1c) in women. In both multivariate and propensity score matching analyses, sleep duration of > 8 h and visceral obestiy showed a positive association in men but a negative association in women with statistical significance. In conclusion, our large-scale cross-sectional study in East Asia identified various gender-specific factors associated with visceral obesity including the long sleep duration.

## Introduction

Visceral obesity is an important risk factor for various conditions such as cardiovascular diseases, type 2 diabetes, and certain types of cancer. First, visceral fat accumulation leads to insulin resistance and type 2 diabetes^1^. In addition, hypertriglyceridemia, low levels of high-density lipoprotein cholesterol (HDL-C), and an increased proportion of small dense low-density lipoprotein cholesterol (LDL-C) phenotypes are important aspects of dyslipidemia observed in patients with visceral obesity^2,3^. Furthermore, an association between visceral obesity and hypertension has been reported^4,5^. Insulin resistance, atherogenic dyslipidemia, and hypertension, all contribute to increased risk of cardiovascular diseases and metabolic syndrome^6–11^. In addition to the abovementioned lifestyle-related diseases, visceral obesity can increase the risk of developing certain types of cancers^12^, especially colorectal^13–15^, esophageal^16^, prostate^17^, and breast cancers^18^. Furthermore, visceral obesity contributes to the development of obstructive sleep apnea (OSA) owing to reduced chest compliance and airway obstruction^19,20^. Interestingly, it has been reported that OSA increases susceptibility to accumulating visceral fat by reducing sleep duration^21^.

Thus, visceral obesity is a significant risk factor for multiple lifestyle-related diseases; hence, identifying its risk factors is essential for prevention. In this study, we performed computed tomography (CT) to calculate the visceral fat area (VFA). We defined visceral obesity as VFA of ≥ 100 cm^2^ because the number of obesity-related disorders increases proportionally to VFA and the average number of such disorders is > 1.0 at a VFA of 100 cm^2^^22^. Obesity is usually evaluated based on body mass index (BMI), but it is well known that BMI shows little correlation with VFA due to wide individual differences. Consequently, BMI is not an accurate marker of visceral obesity, and instead, CT tests are recommended for evaluation^22–24^.

It has been reported that age, gender, genetics, ethnicity, lifestyle, nutritional factors, and hormones (such as sex, corticosteroid, and growth hormones) have correlations with visceral obesity^25^. In addition to these factors, we also focused on the association of sleep duration with the development of visceral obesity. Numerous epidemiological studies in Europe, the United States, and Australia have shown that sleep deprivation is associated with obesity^26–28^. This sleep-obesity relationship is related to changes in dietary intake mediated by an altered balance of appetite-related hormones leptin and ghrelin due to sleep deprivation^29^. Other explanations, such as altered thermoregulation, increased stress hormone levels, and lower physical activity levels, have been suggested^30^. However, to the best of our knowledge, there have been few studies focusing on the association between sleep duration and visceral obesity in East Asia. This large cross-sectional study aimed to clarify the background factors for the development of visceral obesity with a focus on the influence of sleep duration, in Japanese adults.

## Methods

### Participants

We collected 283,397 medical checkup datasets from Kameda Medical Center Makuhari (Chiba-shi, Chiba, Japan) from 2008 to 2020. If a participant had more than one health checkup, all were used independently. Among the available cases, 7863 participants underwent CT to calculate VFA. We excluded participants with missing data for any of the following 11 items: age, BMI, systolic blood pressure (SBP), glycated hemoglobin (HbA1c), HDL-C, LDL-C, triglyceride (TG), uric acid (UA), smoking status, alcohol consumption status, and sleep duration. Finally, 2538 participants were included in our cross-sectional analyses, consisting of 1918 men and 620 women. All participants included in this study underwent blood chemistry tests and CT imaging and were required to complete a questionnaire.

### Questionnaires

The questionnaires contained questions on smoking habits, alcohol consumption, and sleep duration. We analyzed the answers to the following three questions: (i) “Do you have a habit of drinking?” (ii) “Do you have a habit of smoking?” and (iii) “How long do you sleep on an average?” The answers to the question (i) were selected from three categories (rarely, sometimes, and daily). The answers to the question (ii) were selected from three classifications (current smoker, past smoker, and non-smoker). The answers to the question (iii) were categorized into five groups as ordinal variables: < 5-h sleep, 5–6-h sleep, 6–7-h sleep, 7–8-h sleep, and ≥ 8-h sleep.

### VFA measurement using CT

CT cross-sectional scans were obtained at the level of the umbilicus (L3–L4 vertebrae level or L4–L5 vertebrae level). Based on the CT values, the workstation set the boundary lines between the visceral fat and subcutaneous fat and calculated the VFA. The region with CT values of − 125 to − 75 HU was defined as the visceral fat region. The boundary lines were manually adjusted by technicians if needed. We used the CT scanner “Aquilion CXL (64 slices; Canon Medical Systems Asia Pte Ltd, Tochigi, Japan)”: tube voltage 120 kV, tube current 20 mA, thickness 2 mm, CTDI vol 3.3 mGy, DLP 14.7 mGy*cm, effective radiation dose 0.22 mSv, rotation time 0.5 s/rot, helical pitch 11, pitch 0.688. The workstation used was “AZE Virtual Place,” which applied sinogram affirmed iterative reconstruction techniques to analyze CT images.

### Statistical analysis

We conducted univariate and multiple logistic regression analyses using the JMP Pro 16 statistical software program (SAS Institute Inc., Cary, NC, USA) to evaluate the association between candidate background factors and visceral obesity. Visceral obesity was defined as VFA of ≥ 100 cm^2^. We performed subsequent analyses stratified by gender because the relationship between visceral obesity and background factors differed between men and women.

Eleven factors were used as explanatory variables: age, BMI, SBP, HDL-C, TG, UA, HbA1c, LDL-C, smoking status, alcohol consumption status, and sleep duration. The selection of background factors, except for sleep duration, was based on the existing knowledge of the correlation between these risk factors and visceral obesity. Previous research has shown that BMI, hypertension, diabetes, hyperlipidemia, hyperuricemia, smoking, and alcohol consumption are associated with VFA^1–7,24,31–33^. We added “sleep duration” to the list of risk factors, for which reports are limited from East Asia.

First, univariate analysis was conducted using the Mann–Whitney U test for continuous variables and the Pearson chi-square test for categorical variables. Each explanatory factor was categorized as follows: < 18.5 kg/m^2^ (underweight), 18.5–25 kg/m^2^ (normal range), and ≥ 25 kg/m^2^ (overweight) for BMI; < 140 mmHg and ≥ 140 mmHg for SBP; < 6.5% and ≥ 6.5% for HbA1c; < 40 mg/dl and ≥ 40 mg/dl for HDL-C; < 140 mg/dl and ≥ 140 mg/dl for LDL-C; < 150 mg/dl and ≥ 150 mg/dl for TG; < 7 mg/dl and ≥ 7 mg/dl for UA; current smoker, past smoker and non-smoker for smoking status; rarely drinking, sometimes drinking, and daily drinking for alcohol consumption; and < 5, 5–6, 6–7, 7–8, and ≥ 8 h for sleep duration. BMI, SBP, LDL-C, TG, and UA were grouped based on the upper reference limit. HbA1c levels were categorized based on the cutoff point for the diagnosis of diabetes. HDL-C levels were categorized based on the lower reference limit.

In addition, multiple logistic regression analyses were performed to calculate odds ratios (ORs) and 95% confidence intervals (95% CI) and evaluate the relationship between visceral obesity and background factors. Age, BMI, SBP, HbA1c, HDL-C, LDL-C, TG, and UA were analyzed as continuous variables, whereas smoking status, alcohol drinking status, and sleep duration were evaluated as categorical variables. We followed standard methods to estimate the sample size for multiple logistic regression, with at least ten outcomes required for each included independent covariate. We needed 110 participants to appropriately perform multiple logistic regression with 11 covariates and collected 2538 datasets (1147 men and 131 women for VFA ≥ 100 cm^2^; 771 men and 489 women for VFA < 100 cm^2^). The variance inflation factor was used to check for multicollinearity. The chi-square goodness-of-fit test and Hosmer–Lemeshow test were conducted to evaluate the fitness of the model. Statistical significance was set at a p-value of < 0.05.

Finally, we performed logistic regression analyses using each sleep duration group (< 5, 5–6, 6–7, 7–8, and ≥ 8 h) and their respective propensity scores (PS) as covariates to estimate the precise sleep-obesity relationship by eliminating the small sample bias and reducing the effects of outliers. These analyses were conducted using the JMP Pro 16 statistical software (SAS Institute Inc.). Each PS was estimated with a logistic regression model using age, BMI, SBP, HbA1c, HDL-C, LDL-C, TG, UA, smoking status, alcohol drinking status, and other sleep duration groups. In the PS-adjusted logistic regression models, ORs and 95% CIs were calculated considering 6–7-h sleep as a reference. Statistical significance was set at a p-value of < 0.05.

### Ethics approval and consent to participate

This study was conducted in accordance with the World Medical Association’s Declaration of Helsinki. Written informed consent was obtained from all the study participants. The study was also approved by the ethics committee of the Kameda Medical Center (No. 17-075) and the University of Tokyo (No. 2865). All data were fully anonymized before the analysis.

## Results

### Characteristics of study participants

**Figure 1:**
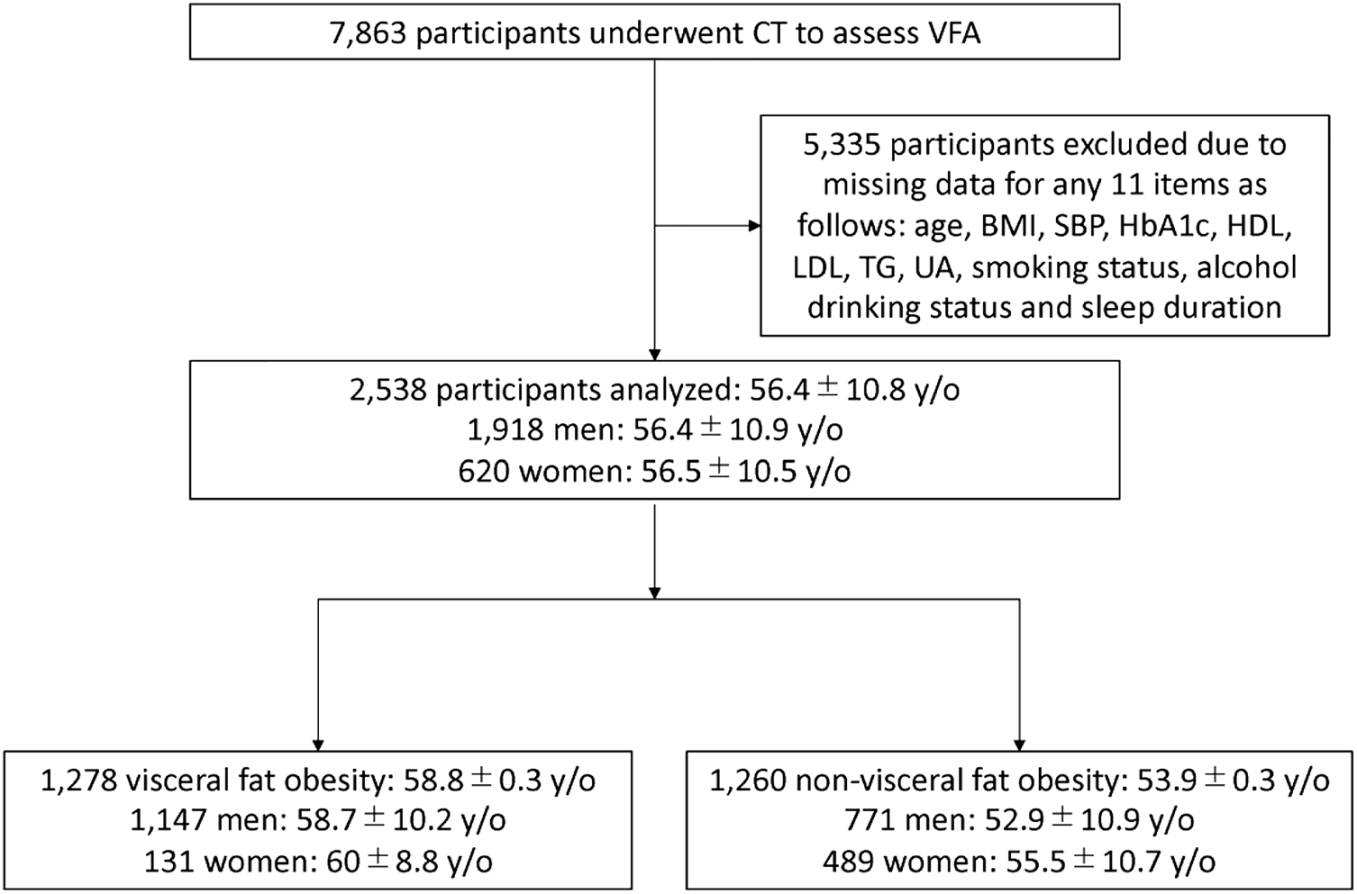
Study recruitment flowchart. Of 7863 participants who underwent computed tomography (CT) to assess the visceral fat area (VFA), 2538 participants with no missing data for age, body mass index (BMI), systolic blood pressure (SBP), glycated hemoglobin (HbA1c), high-density lipoprotein (HDL) and low-density lipoprotein (LDL) levels, triglyceride (TG) levels, uric acid (UA), smoking status, alcohol consumption status, and sleep duration were analyzed. Age is expressed as mean ± standard deviation.

Of the 283,397 medical checkup datasets, we selected 7863 cases that underwent CT to evaluate VFA. From the 7863 participants, we excluded participants missing at least one item among age, BMI, SBP, HbA1c, HDL-C, LDL-C, TG, UA, smoking status, alcohol intake status, and sleep duration (Fig. 1). The remaining 2538 participants consisted of 1918 men and 620 women with a mean age of 56.4 ± 10.8 years (Table 1). Among them, 1147 men (59.8%) and 131 women (21.1%) were observed to have visceral obesity. Mean VFA was 116.5 ± 47.1 cm^2^ in men and 70.0 ± 41.2 cm^2^ in women (Table 1).

**Table 1:** Characteristics of the study participants.

Variable	All subjects (n = 2538), N (%)	Men (n = 1918), N (%)	Women (n = 620), N (%)	*P* value	
**Age, y/o**					
Mean ± SD	56.4 ± 10.8	56.4 ± 10.9	56.5 ± 10.5	0.96	¶
< 30	12 (0.5)	9 (0.5)	3 (0.5)	0.46	∆
30–40	140 (5.5)	115 (6.0)	25 (4.0)		
40–50	565 (22.3)	426 (22.2)	139 (22.4)		
50–60	810 (31,9)	600 (31.3)	210 (33.9)		
60–70	709 (27.9)	542 (28.3)	167 (26.9)		
≥ 70	302 (11.9)	226 (11.8)	76 (12.3)		
**BMI, kg/m**^2^					
Mean ± SD	24.5 ± 3.4	25.0 ± 3.2	22.8 ± 3.6	< 0.001*	¶
< 18.5	61 (2.4)	8 (0.4)	53 (8.6)	< 0.001*	∆
18.5–25	1458 (57.4)	1041 (54.3)	417 (67.3)		
≥ 25	1019 (40.2)	869 (45.3)	150 (24.2)		
**VFA, cm**^**2**^					
Mean ± SD	105.1 ± 49.9	116.5 ± 47.1	70.0 ± 41.2	< 0.001*	¶
≥ 100	1278 (50.4)	1147 (59.8)	131 (21.1)	< 0.001*	∆
< 100	1260 (49.6)	771 (40.2)	489 (78.9)		
**SBP, mmHg**					
Mean ± SD	119.6 ± 15.0	120.7 ± 14.0	116.3 ± 17.2	< 0.001*	¶
≥ 140	228 (9.0)	171 (8.9)	57 (9.2)	0.83	∆
< 140	2310 (91.0)	1747 (91.1)	563 (90.8)		
**HbA1c, %**					
Mean ± SD	5.82 ± 0.71	5.84 ± 0.75	5.76 ± 0.53	0.26	¶
≥ 6.5	256 (10.1)	209 (10.9)	47 (7.6)	0.017*	∆
< 6.5	2282 (89.9)	1709 (89.1)	573 (92.4)		
**HDL-C, mg/dL**					
Mean ± SD	60.7 ± 16.3	57.5 ± 14.9	70.6 ± 16.5	< 0.001*	¶
< 40	142 (5.6)	136 (7.1)	6 (0.9)	< 0.001*	∆
≥ 40	2396 (94.4)	1782 (92.9)	614 (99.4)		
**LDL-C, mg/dL**					
Mean ± SD	128.3 ± 29.9	127.3 ± 29.2	131.3 ± 32.0	0.019*	¶
≥ 140	853 (33.6)	620 (32.3)	233 (37.6)	0.016*	∆
< 140	1685 (66.4)	1298 (67.7)	387 (62.4)		
**TG, mg/dL**					
Mean ± SD	133.5 ± 112.4	143.4 ± 122.2	102.6 ± 65.7	< 0.001*	¶
≥ 150	712 (28.1)	615 (32.1)	97 (15.6)	< 0.001*	∆
< 150	1826 (71.9)	1303 (67.9)	523 (84.4)		
**UA, mg/dL**					
Mean ± SD	5.89 ± 1.34	6.2 ± 1.25	4.9 ± 1.12	< 0.001*	¶
≥ 7	550 (21.7)	522 (27.2)	28 (4.5)	< 0.001*	∆
< 7	1988 (78.3)	1396 (72.8)	592 (95.5)		
**Sleep duration, h**					
Mean ± SD	6.15 ± 1.07	6.18 ± 1.06	6.05 ± 1.08	0.004*	¶
< 5	162 (6.4)	127 (6.6)	35 (5.7)	0.001*	∆
5–6	625 (24.6)	434 (22.6)	191 (30.8)		
6–7	997 (39.2)	768 (40.0)	229 (36.9)		
7–8	530 (20.9)	410 (21.4)	120 (19.4)		
≥ 8	224 (8.8)	179 (9.3)	45 (7.3)		
**Smoking status**					
Current smoker	502 (19.8)	463 (24.1)	39 (6.3)	< 0.001*	∆
Past smoker	841 (33.1)	755 (39.4)	86 (13.9)		
Non smoker	1195 (47.1)	700 (36.5)	495 (79.8)		
**Alcohol drinking status**					
Rarely drinking	837 (33.0)	504 (26.3)	333 (53.7)	< 0.001*	∆
Sometimes drinking	926 (36.5)	714 (37.2)	212 (34.2)		
Daily drinking	775 (30.5)	700 (36.5)	75 (12.1)		

*P*-values were calculated between men and women.

*Statistically significant p-values, ¶ Mann–Whitney U test, ∆ Pearson chi-square test.

Mean BMI, VFA, SBP, HDL-C, LDL-C, TG, UA, and sleep duration showed significant differences between men and women. Except for the mean HDL-C and LDL-C levels, all other parameters were higher among men. Specifically, the percentage of participants with HbA1c of ≥ 6.5%, smokers, and drinkers were remarkably higher among men than among women (Table 1).

### Univariately associated factors with visceral obesity

Univariate analyses were independently performed for both men and women to estimate the association of the 11 background factors with visceral obesity (Table 2). The correlation of age, BMI, SBP, HbA1c, HDL-C, TG, UA, smoking status, alcohol consumption status, and sleep duration with visceral obesity were statistically significant among men. Men with visceral obesity were older, smoked, consumed alcohol, and had a higher BMI, SBP, HbA1c, TG, UA, and lower HDL-C. On the other hand, the correlation of age, BMI, SBP, HbA1c, HDL-C, LDL-C, TG, and UA were significant among women. Likewise, women with visceral obesity were more likely to be older and had a higher BMI, SBP, HbA1c, LDL-C, TG, UA, and a lower HDL-C. Consequently, all 11 factors that were analyzed univariately were significantly associated with visceral obesity, at least either in men or in women; therefore, we focused on all variables for multiple analyses.

**Table 2:** Univariate analysis of background factors contributing to visceral obesity.

Variable	Men (n = 1918), N (%)				Women (n = 620), N (%)		
	Visceral obesity	Non-obesity	*P* value		Visceral obesity	Non-obesity	*P* value
**Age, y/o**							
Mean ± SD	58.7 ± 10.2	52.9 ± 10.9	< 0.001*	¶	60 ± 8.8	55.5 ± 10.7	< 0.001*
< 30	3 (0.3)	6 (0.8)	< 0.001*	∆	0 (0)	3 (0.6)	< 0.001*
30–40	33 (2.9)	82 (10.6)			1 (0.7)	24 (4.9)	
40–50	187 (16.3)	239 (31.0)			13 (9.9)	126 (25.8)	
50–60	394 (34.5)	206 (26.7)			49 (37.4)	161 (32.9)	
60–70	350 (30.5)	192 (24.9)			46 (35.1)	121 (24.7)	
≥ 70	180 (15.7)	46 (6.0)			22 (16.8)	54 (11.0)	
**BMI, kg/m**^**2**^							
Mean ± SD	26.2 ± 3.2	23.4 ± 2.4	< 0.001*	¶	25.9 ± 3.3	21.9 ± 3.2	< 0.001*
< 18.5	0 (0)	8 (1.0)	< 0.001*	∆	0 (0)	53 (10.8)	< 0.001*
18.5–25	454 (39.6)	587 (76.1)			59 (45.0)	358 (73.2)	
≥ 25	693 (60.4)	176 (22.8)			72 (55.0)	78 (16.0)	
**SBP, mmHg**							
Mean ± SD	122.8 ± 13.6	117.5 ± 14.1	< 0.001*	¶	126 ± 14.4	113.7 ± 17.0	< 0.001*
≥ 140	115 (10.0)	56 (7.3)	0.037*	∆	19 (14.5)	38 (7.8)	0.018*
< 140	1032 (90.0)	715 (92.7)			112 (85.5)	451 (92.2)	
**HbA1c, %**							
Mean ± SD	5.96 ± 0.86	5.67 ± 0.51	< 0.001*	¶	6.1 ± 0.68	5.67 ± 0.44	< 0.001*
< 6.5	975 (85.0)	734 (95.2)	< 0.001*	∆	102 (77.9)	471 (96.3)	< 0.001*
≥ 6.5	172 (15.0)	37 (4.8)			29 (22.1)	18 (3.7)	
**HDL-C, mg/dL**							
Mean ± SD	55.1 ± 13.5	61.2 ± 16.0	< 0.001*	¶	59.7 ± 13.3	73.5 ± 16.0	< 0.001*
< 40	101 (8.8)	35 (4.5)	< 0.001*	∆	5 (3.8)	1 (0.2)	< 0.001*
≥ 40	1046 (91.2)	736 (95.5)			126 (96.2)	488 (99.8)	
**LDL-C, mg/dL**							
Mean ± SD	127.4 ± 29.6	127.1 ± 28.7	0.62	¶	141.7 ± 32.3	128.5 ± 31.3	< 0.001*
≥ 140	766 (66.8)	532 (69.0)	0.31	∆	65 (49.6)	322 (65.9)	< 0.001*
< 140	381 (33.2)	239 (31.0)			66 (50.4)	167 (34.1)	
**TG, mg/dL**							
Mean ± SD	159.8 ± 135.0	119.1 ± 95.1	< 0.001*	¶	159.4 ± 85.3	87.4 ± 49.4	< 0.001*
≥ 150	464 (40.5)	151 (19.6)	< 0.001*	∆	56 (42.8)	41 (8.4)	< 0.001*
< 150	683 (59.6)	620 (80.4)			75 (57.2)	448 (91.6)	
**UA, mg/dL**							
Mean ± SD	6.36 ± 1.22	5.97 ± 1.25	< 0.001*	¶	5.59 ± 1.16	4.74 ± 1.04	< 0.001*
≥ 7	351 (30.6)	171 (22.2)	< 0.001*	∆	15 (11.5)	13 (2.7)	< 0.001*
< 7	796 (69.4)	600 (77.8)			116 (88.6)	476 (97.3)	
**Sleep duration, h**							
Mean ± SD	6.23 ± 1.13	6.10 ± 0.96	0.055	¶	5.96 ± 0.98	6.07 ± 1.1	0.32
< 5	79 (6.9)	48 (6.2)	< 0.001*	∆	5 (3.8)	30 (6.1)	0.17
5–6	249 (21.7)	185 (24.0)			49 (37.4)	142 (29.0)	
6–7	445 (38.8)	323 (41.9)			45 (34.4)	184 (37.6)	
7–8	233 (20.3)	177 (23.0)			27 (20.6)	93 (19.0)	
≥ 8	141 (12.3)	38 (4.9)			5 (3.8)	40 (8.2)	
**Smoking status**							
Current smoker	286 (24.9)	177 (23.0)	< 0.001*	∆	11 (8.4)	28 (5.7)	0.5
Past smoker	474 (41.3)	281 (36.5)			19 (14.5)	67 (13.7)	
Non smoker	387 (33.7)	313 (40.6)			101 (77.1)	394 (80.6)	
**Alcohol drinking status**							
Rarely drinking	300 (26.2)	204 (26.5)	0.011*	∆	77 (58.8)	256 (52.4)	0.19
Sometimes drinking	400 (34.9)	314 (40.7)			36 (27.5)	177 (36.0)	
Daily drinking	447 (39.0)	253 (32.8)			18 (13.7)	57 (11.7)	

*Statistically significant p-values, ¶ Mann–Whitney U test, ∆ Pearson chi-square test.

### Assessment of the association between background factors and visceral obesity by multiple logistic regression

Using the aforementioned background factors, we conducted multiple logistic regression analyses on men and women (Table 3). Standardized coefficients (β), odds ratios (OR), and p-values were calculated. In addition, the chi-square goodness-of-fit and Hosmer–Lemeshow tests were performed, and the fitness of the model was evaluated (chi-square test, p < 0.0001; Hosmer–Lemeshow test, p = 0.8543).

**Table 3:** Multiple logistic regression analyses assessing the association between visceral obesity and the background factors.

Variable	Men			Women		
	Standardized coefficient	Odds ratio (95% confidence interval)	*P* value	Standardized coefficient	Odds ratio (95% confidence interval)	*P* value
Age	0.88	2.4 (2.09–2.76)	< 0.001*	0.43	1.54 (1.12–2.1)	0.007*
BMI	1.3	3.68 (3.11–4.38)	< 0.001*	1.12	3.07 (2.18–4.31)	< 0.001*
SBP	0.06	1.06 (0.94–1.2)	0.3	−0.05	0.95 (0.7–1.28)	0.73
HbA1c	0.14	1.15 (1.0–1.33)	0.056	0.36	1.43 (1.07–1.92)	0.016*
HDL-C	−0.29	0.75 (0.65–0.86)	< 0.001*	−0.17	0.84 (0.58–1.22)	0.37
LDL-C	0.05	1.05 (0.94–1.18)	0.38	−0.01	0.99 (0.77–1.28)	0.95
TG	0.18	1.2 (1.02–1.43)	0.034*	0.89	2.42 (1.71–3.45)	< 0.001*
UA	0.33	1.39 (1.23–1.57)	< 0.001*	0.27	1.31 (0.99–1.73)	0.063
**Smoking status**						
Current smoker	0.08	1.08 (0.96–1.22)	0.21	-0.01	0.99 (0.85–1.14)	0.87
Past smoker	0.1	1.11 (0.98–1.25)	0.1	0.05	1.05 (0.92–1.2)	0.5
Non smoker	Reference	Reference	Reference	Reference	Reference	Reference
**Alcohol drinking status**						
Rarely drinking	Reference	Reference	Reference	Reference	Reference	Reference
Sometimes drinking	−0.09	0.92 (0.8–1.05)	0.21	0.03	1.03 (0.88–1.21)	0.7
Daily drinking	0.15	1.16 (1.01–1.34)	0.039*	0.08	1.08 (0.93–1.25)	0.3
**Sleep duration, h**						
**< 5**	0.05	1.05 (0.94–1.17)	0.4	−0.04	0.96 (0.83–1.1)	0.53
**5–6**	−0.02	0.98 (0.87–1.09)	0.68	0.07	1.07 (0.91–1.26)	0.41
**6–7**	Reference	Reference	Reference	Reference	Reference	Reference
**7–8**	−0.01	0.99 (0.89–1.11)	0.88	−0.01	0.99 (0.85–1.16)	0.91
**≥ 8**	0.17	1.19 (1.06–1.34)	0.004*	−0.18	0.83 (0.7–0.98)	0.031*

Multiple logistic regression analyses were performed. * p-values indicate statistically significant.

For men, factors that were significantly associated with visceral obesity were age [β = 0.88, OR = 2.4 (2.09–2.76), p < 0.0001], BMI [β = 1.3, OR = 3.68 (3.11–4.38), p < 0.0001], HDL-C [β =  − 0.29, OR = 0.75 (0.65–0.86), p < 0.0001], TG [β = 0.18, OR = 1.2 (1.02–1.43), p = 0.0343], UA [β = 0.33, OR = 1.39 (1.23–1.57), p < 0.0001], daily drinking [β = 0.15, OR = 1.16 (1.01–1.34), p = 0.0385], and ≥ 8-h sleep [β = 0.17, OR = 1.19 (1.06–1.34), p = 0.0044]. Meanwhile, for women, the following factors were significantly associated: age [β = 0.43, OR = 1.54 (1.12–2.1), p = 0.0065], BMI [β = 1.12, OR = 3.07 (2.18–4.31), p < 0.0001], HbA1c [β = 0.36, OR = 1.43 (1.07–1.92), p = 0.016], TG [β = 0.89, OR = 2.42 (1.71–3.45), p < 0.0001], and ≥ 8-h sleep [β =  − 0.18, OR = 0.83 (0.7–0.98), p = 0.0306].

Therefore, age, BMI, and TG level were significantly associated with visceral obesity, regardless of gender. On the other hand, sleep duration of > 8 h and visceral obesity showed a positive association in men but a negative association in women with both statistical significance. High UA levels, low HDL-C levels, and daily alcohol consumption were significantly associated with visceral obesity in men. In contrast, higher HbA1c levels were significantly associated with visceral obesity in women.

### Sleep duration and visceral obesity relationship

**Figure 2:**
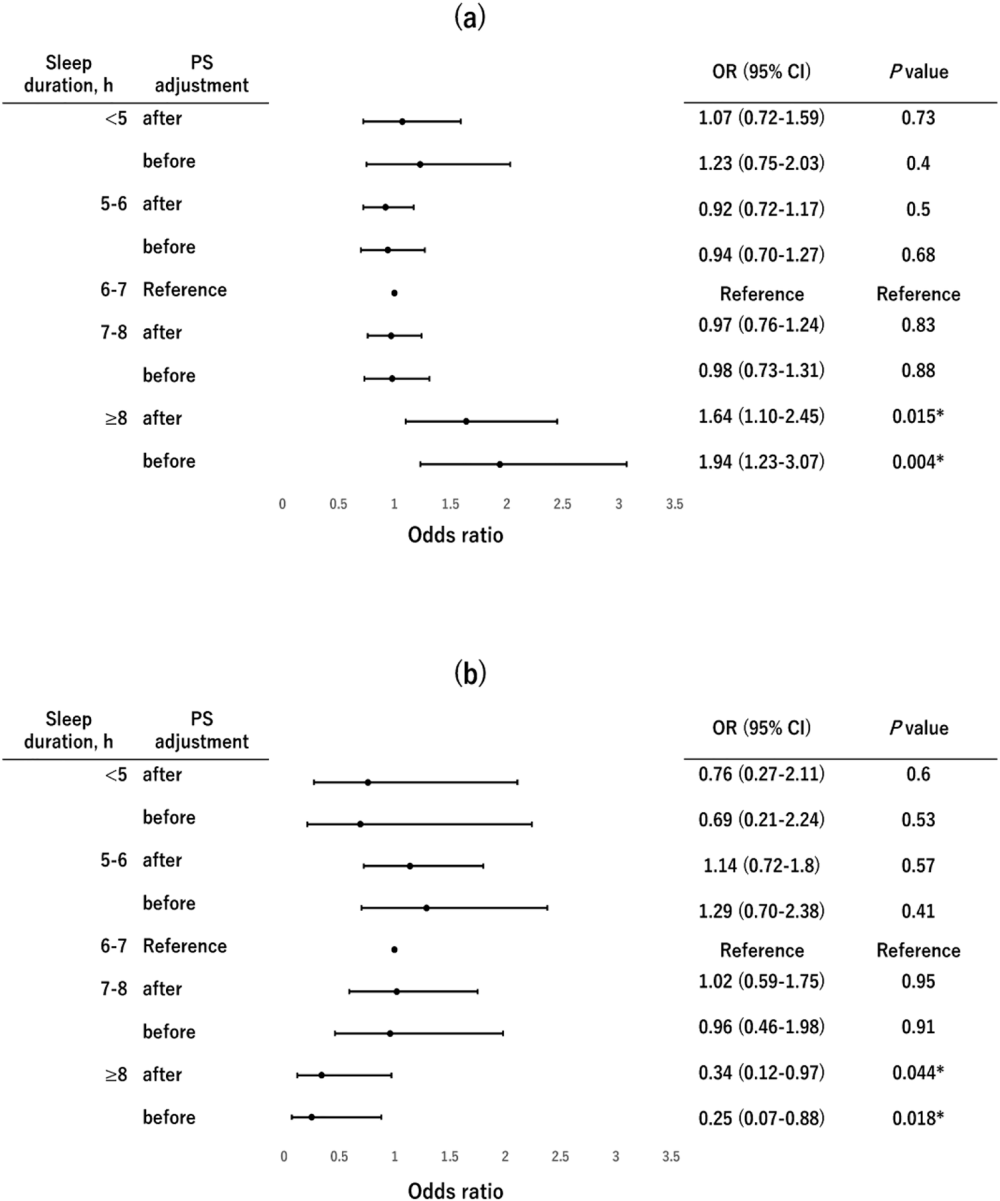
Forest plots of odds ratios and 95% confidence intervals before and after propensity score adjustment. Forest plots of the odds ratios (OR) and 95% confidence intervals (CI) of each sleep duration group for visceral obesity in men (**a**) and women (**b**). Sleep duration was categorized as follows: < 5, 5–6, 6–7, 7–8, and ≥ 8 h. OR and 95% CI were calculated using logistic regression models both before and after propensity score (PS) adjustment. The 6–7 h sleep group was taken as a reference for both men and women.

We evaluated the gender-specific sleep-obesity relationship in the logistic regression model adjusted for age, BMI, SBP, HbA1c, HDL-C, LDL-C, TG, UA, smoking status, and alcohol consumptionstatus using each sleep duration group (< 5, 5–6, 6–7, 7–8, and ≥ 8 h) and respective PSs as covariates. We compared OR, 95% CI, and p-values calculated using the logistic regression model both before and after PS adjustments (Fig. 2). The logistic regression model adjusted for other background factors revealed remarkable differences in the sleep-obesity relationship by gender. Long sleep (≥ 8 h) and visceral obesity showed a positive association in men with statistical significance (OR (95% CI) = 1.64 (1.1–2.45), p = 0.015; Fig. 2a). In contrast, they showed a negative association in women with statistical significance (OR (95% CI) = 0.34 (0.12–0.97), p = 0.044; Fig. 2b). Thus, the relationship of long sleep and visceral obesity remarkably differed between men and women. On the other hand, sleep deprivation (< 5 h) and visceral obesity showed no significant association both in men (OR (95% CI) = 1.07 (0.72–1.59), p = 0.73; Fig. 2a) and in women (OR (95% CI) = 0.76 (0.27–2.11), p = 0.60; Fig. 2b).

## Discussion

Age and blood pressure are common factors associated with visceral obesity in Europe and the United States^25,34–39^. Aging has been reported to result in a reduction of fat-free mass and muscle mass and an increase in visceral fat^35–37^. In addition, although the correlation of BMI with visceral obesity has been demonstrated, BMI alone cannot effectively evaluate visceral obesity and the various risks associated with it^22–24^. Our large cross-sectional study from East Asia showed that higher age and higher BMI were significantly associated with visceral obesity in both genders (Table 3), which is consistent with previous reports^25,34^. On the other hand, SBP was not associated with visceral obesity, regardless of gender (Table 3). This difference could be attributed to our study design because we did not account for the possibility that participants were being administered antihypertensive medications. To evaluate the precise association, the influence of antihypertensive medications should be considered in future studies.

Previous studies in Europe and the United States have shown that high HbA1c levels, dyslipidemia, and high UA level are associated with visceral obesity^1–3,32,40^. In terms of HbA1c, visceral obesity is associated with insulin resistance and type 2 diabetes^1^. Dyslipidemia, especially high TG and low HDL-C levels, is associated with visceral obesity^2,3,40^. Regarding LDL-C, a previous study showed that in patients who were obese, LDL-C levels were frequently normal, but small dense LDL-C phenotypes were increased^41^. Our study on Japanese adults revealed a positive association between high HbA1c and visceral obesity, in both men and women (Table 3), which is similar to a previous report^1^. As for dyslipidemia, high TG level was significantly associated with visceral obesity in both genders, whereas low HDL-C level was associated only with visceral obesity in men (Table 3). The lack of correlation of low HDL-C in women is attributed to the effect of estrogen in increasing HDL-C levels^42^. Regarding LDL-C, a significant positive correlation with visceral obesity was not observed, regardless of gender (Table 3), which is consistent with another previous report^41^. In addition, high UA levels were significantly associated only with visceral obesity in men (Table 3), which has been previously reported^32^. The effect of estrogen in preventing an increase in Ua level is possibly involved in the lack of an association between UA and visceral obesity among women^43^.

As lifestyle-related factors for visceral obesity, smoking, and alcohol use have been the focus of research in Europe and the United States^31,33^. Meanwhile, our large cross-sectional study on Japanese adults showed that smoking status was not associated with visceral obesity regardless of gender, and daily alcohol consumption was significantly associated with visceral obesity only in men (Table 3). As for smoking, the lack of association seen in this study may be attributed to our study design, which did not consider the number of cigarettes smoked per day. In terms of alcohol use, previous research revealed that excess alcohol intake is associated with obesity^31^, but our study did not consider the amount of alcohol consumption.

Sleep duration has been studied as a risk factor for visceral obesity in Europe, the United States, and Australia^26–28^, but there have not been enough studies on the relationship between sleep and obesity in East Asia. Several previous reports have shown that sleep restriction causes visceral obesity^26–30,44–46^. Sleep restriction promotes hormonal changes such as high cortisol, high ghrelin, low sensitivity to leptin, and low melatonin, causing increased food intake, decreased energy expenditure, weight gain, and visceral fat accumulation^44–46^. Obesity-related sleep disorders play an important role in the sleep-obesity relationship^46^. Obesity increases the risk of OSA and decreases the amount and quality of sleep^19,20,46^. In addition, visceral adipose tissue releases certain inflammatory cytokines, such as tumor necrosis factor-α, interleukin (IL)-1, and IL-6, which could play a role in causing sleep disturbance^46^. Furthermore, obesity-promoting high-fat and low-fiber diets cause sleep deprivation^46^. However, it has also been indicated that long sleep duration is associated with visceral obesity^47^. To date, there are not enough mechanical and interventional studies to investigate whether and how long sleep duration confers obesity risks^47^.

Our study, a large cross-sectional study from East Asia, revealed the sleep-obesity relationship both in men and in women (Fig. 2). Logistic regression analyses adjusted for other background factors revealed that the sleep-obesity risk relationship in men was U-shaped (Fig. 2a) whereas that in women was inverted U-shaped (Fig. 2b). Thus, the sleep-obeisty relationship was remarkably different between men and women. Especially, long sleep duration (≥ 8 h) was positively associated with visceral obesity in men, but was negatively associated with visceral obesity in women (Fig. 2). It is speculated that such gender-specific difference might be attributed to the different lifestyle and health consciousness of men and women. We speculated that men with long sleep might be likely to have enough money and be overweight with overnutrition; or that women with high health consciousness might tend to sleep longer so as to keep their weight in the normal range. To focus on this speculation, we need to consider other confounding factors such as income, occupation, diet, etc. in the future.

On the other hand, sleep deprivation (< 5 h) was not significantly associated with visceral obesity both in men and in women. This seems to be inconsistent with previous findings in Europe, the United States, or Australia^26–30,44–46^ , in most of which BMI was used as the criteria of obesity. To evaluate the relationship between obesity and various health-related factors, we believe not BMI but VFA should be used because BMI cannot effectively evaluate visceral obesity and reflect the risk of various diseases^22–24^. In this sense, our study provided the reliable relationship between short sleep and obesity. It is also speculated that the difference of results between our study and previous findings^26–30,44–46^ might be attributed to the difference of genetics and ethnicity between East Asia and Western countries. Multiethnic mixed studies focusing on the relationship between sleep deprivation and VFA should be needed in the future. Our study is novel in that it showed that the sleep-obesity relationship, especially between long sleep and obesity, differs remarkably between men and women. The gender difference in the sleep-obesity association has not been mentioned before, and our research provides new findings in identifying risk factors for visceral obesity.

The study has some limitations to it. The first being its design. Cross-sectional research is a single-point analysis that cannot accurately evaluate the sleep-obesity relationship because obesity is a consequence of several risk factors. However, our study was a large-scale cross-sectional study and provided reliable associations between multiple factors and visceral obesity at a single point in East Asia. The second limitation is that we did not consider whether participants used antihypertensive medications, how much they smoked, how much alcohol they drank, or their quality of sleep. These information should be acquired to accurately estimate the association between each factor and visceral obesity. The third limitation is the possibility of other confounding factors that influence the sleep-obesity relationship, e.g., income, occupation, and diet. The fourth limitation is that the length of sleep was self-reported in the questionnaires. To evaluate sleep duration more objectively, it may be effective measuring the length by such as sleep monitoring devises in the future. The fifth limitation is the possibility of the sampling bias. Especially, the proportion of participants with low body weight (BMI < 18.5) was small (Table1), and that with obesity (BMI ≥ 30) was also small [176 men (9.2% of all male participants), 27 women (4.4% of all female participants)]. This sampling bias may result in the inconsistency between our findings and previous findings from Western conutries^26–30,44–46^ about the short sleep and obesity relationship. The final limitation is that we did not consider the influence of change in menstrual status on the sleep-obesity relationship. The major difference by gender is the sex hormonal differnce. Therefore, in women, the analyses stratified by before/after menopause might suggest new findings revealing background factors influencing on the sleep-obesity relationship more clearly.

Future prospective observational studies should be conducted to evaluate changes in VFA and obesity-associated factors over time. Particularly, in terms of the influence of sleep on visceral obesity, prospective observational studies and randomized control trials are needed to evaluate the sleep-obesity relationship reliably. Intervention studies on sleep deprivation have been conducted^44^, but regarding longer sleep durations, there are not enough intervention studies^47^. In addition, mechanisms involving hormonal changes and changes in cytokine levels should be investigated in the future. Especially, our study suggested differences in the sleep-obesity association by gender, so it is meaningful to conduct further studies focusing on gender differences.

Our findings based on a large cross-sectional study in East Asia contributes to identifying risk factors for visceral obesity. Our study is novel in highlighting that the sleep-obesity relationship differed remarkably between men and women, especially between long sleep and obesity. It showed that sleep duration longer than 8 h was positively associated with visceral obesity with statistical significance in men, but was negatively associated with statistical significance in women. Our study also demonstrated the association between visceral obesity and several other factors, such as age, BMI, and TGs in both genders; low HDL-C, high UA levels, and daily alcohol consumption in men; and high HbA1c in women.

## Data Availability

The datasets generated and/or analyzed during the current study are available from the corresponding author on reasonable request.
